# Examination of MARCO Activity on Dendritic Cell Phenotype and Function Using a Gene Knockout Mouse

**DOI:** 10.1371/journal.pone.0067795

**Published:** 2013-07-01

**Authors:** Hiroshi Komine, Lisa Kuhn, Norimasa Matsushita, James J. Mulé, Shari Pilon-Thomas

**Affiliations:** 1 Immunology Program, H Lee Moffitt Cancer Center and Research Institute, Tampa, Florida, United States of America; 2 Cutaneous Oncology Program, H Lee Moffitt Cancer Center and Research Institute, Tampa, Florida, United States of America; New York University, United States of America

## Abstract

We have reported the upregulation of MARCO, a member of the class A scavenger receptor family, on the surface of murine and human dendritic cells (DCs) pulsed with tumor lysates. Exposure of murine tumor lysate-pulsed DCs to an anti-MARCO antibody led to loss of dendritic-like processes and enhanced migratory capacity. In this study, we have further examined the biological and therapeutic implications of MARCO expression by DCs. DCs generated from the bone marrow (bm) of MARCO knockout (MARCO^-/-^) mice were phenotypically similar to DCs generated from the bm of wild-type mice and produced normal levels of IL-12 and TNF-α when exposed to LPS. MARCO^-/-^ DCs demonstrated enhanced migratory capacity in response to CCL-21 *in vitro*. After subcutaneous injection into mice, MARCO^-/-^ TP-DCs migrated more efficiently to the draining lymph node leading to enhanced generation of tumor-specific IFN-γ producing T cells and improved tumor regression and survival in B16 melanoma-bearing mice. These results support targeting MARCO on the surface of DCs to improve trafficking and induction of anti-tumor immunity.

## Introduction

Dendritic cells (DCs) play a pivotal role in the induction of anti-tumor immunity. Immunization approaches employing DCs pulsed with tumor lysates or apoptotic tumor cells have been examined in murine models [1–5]. Vaccination with tumor antigen-pulsed DCs can induce anti-tumor T cell proliferation, cytotoxicity, and regression of established tumors. Vaccination with tumor antigen-pulsed DCs has been shown to be safe and effective at inducing anti-tumor T cell responses in patients with advanced cancers [6,7].

We have previously demonstrated upregulated macrophage receptor with collagenous structure (MARCO) expression in DCs pulsed with LPS or tumor lysates [8]. MARCO is a member of the class-A scavenger receptor family and binds to Gram-positive and negative bacteria [9]. MARCO was initially characterized on macrophages in the marginal zone of the spleen and in the medullary cord of the lymph nodes [10]. MARCO has been shown to be important for immune responses to bacterial infections by mediating the binding and phagocytosis of pathogens. MARCO has also been implicated in the formation of lamellipodia-like structures and dendritic processes in macrophages and dendritic cells [11,12]. In this study, we further examined the role of MARCO in DCs. We have developed MARCO knockout mice and examined the phenotype and efficacy of DCs generated from the bone marrow of these mice at inducing anti-tumor immunity in a murine B16 melanoma model.

## Materials and Methods

### Animals

Six-to twelve-week-old female C57BL/6 mice were purchased from Charles River Laboratories, Inc. (Wilmington, MA) and Harlan Laboratory (Indianapolis, IN). MARCO-deficient mice (MARCO^-/-^) were developed on the C57BL/6 background in our laboratory. This study was carried out in strict accordance with the recommendations in the Guide for the Care and Use of Laboratory Animals of the National Institutes of Health. The protocol was reviewed and approved by the Institutional Animal Care and Use Committee at the University of South Florida (# A4100-01). Mice were humanely euthanized by CO_2_ inhalation according to the American Veterinary Medical Association Guidelines. Mice were observed daily and humanely euthanized if a solitary subcutaneous tumor exceeded 1.5 cm in diameter or mice showed signs referable to metastatic cancer. All mice were maintained in the Animal Research Facility at the H. Lee Moffitt Cancer Center and Research Institute.

### PCR

To perform PCR, tail snips were collected from mice that were less than 21 days old. DNA from tail snips was isolated with Wizard SV Genomic DNA Purification System (Promega). The genotype of MARCO^-/-^ mice was confirmed by PCR using the following primers:

MARCO-F: 5’ GGT TGG TTT GGT GGC TAT GTA GAG 3’ (Intron 13)

MARCO-R: 5’ CCG GAC GCG TTG GAA AGA TT 3’ (Exon 16)

neo: 5’ CAA AAC CAC ACT GCT CGA CA 3’ (*neo* ^*r*^).

MARCO-F and MARCO-R primers were used to verify the WT allele. MARCO-F and neo primers were used to verify the targeted allele. Either combination of primers results in a 2.0 kbp PCR product.

### Reverse Transcriptase Polymerase Chain Reaction (RT-PCR)

For detection of MARCO mRNA, DC were unpulsed, pulsed with 1 µg/ml LPS (from *E. coli* 0111: B4; Sigma), or B16 tumor lysate for 24 hours. To isolate mRNA from DC, RNeasy Micro Kit (Qiagen, Valencia, CA) was used according to the supplier’s instructions. For RT-PCR reactions, 100 ng mRNA was used to synthesize cDNA with Ready-to-Go RT-PCR beads (Amersham Biosciences, Buckinghamshire, England). The cDNA synthesis reaction was performed at 37°C for 60 min followed by 95°C for 5 min. After the cDNA reaction, 400 nM primers were added into the reaction mixture. The following primers were used for murine MARCO PCR reactions; Sense: 5’-GCA CTG CTG CTG ATT CAA GTT C-3’, Anti-sense: 5’-AGT TGC TCC TGG CTG GTA TG-3’ (205 bp product).

### Cell Line and Culture Medium

The B16-BL6 (denoted B16) melanoma cell line was derived from a spontaneous melanoma in a C57BL/6 mouse and is considered poorly immunogenic [13]. B16 was cultured in complete medium (CM) and maintained by serial *in vitro* passage. CM consists of RPMI1640 medium (Mediatech, Inc., Herndon, VA) supplemented by 10% heat-inactivated fetal bovine serum (Atlanta Biologicals, Lawrenceville, GA), 1 mM sodium pyruvate (Mediatech, Inc.), 0.1 mM non-essential amino acids (Mediatech, Inc.), 100 units/ml penicillin, 100 µg/ml streptomycin (Mediatech, Inc.), 50 µM 2-mercaptoethanol (Sigma, St. Louis, MO), 0.5 µg/ml fungizone (Cambrex, Walkersville, MD), and 10 µg/ml gentamicin (Cambrex).

### Generation of DC and Pulsing with Tumor Lysate

Bone marrow cells were collected from the femurs and tibias of C57BL/6 wild-type and MARCO^-/-^ mice under sterile conditions. Erythrocytes were lysed with ACK lysing buffer (0.15 M NH_4_Cl, 1 mM KHCO_3_, and 0.1 mM EDTA in sterile water). Erythrocyte-depleted bone marrow cells were then washed twice with Dulbecco’s phosphate-buffered saline (PBS) (Mediatech, Inc.) and resuspended in CM containing 20 ng/ml of recombinant mouse granulocyte/macrophage colony-stimulating factor (GM-CSF) and recombinant mouse interleukin-4 (IL-4) (both from R&D Systems, Minneapolis, MN) at the concentration 1 x 10^6^ cells/ml, and then incubated at 37^o^C and 5% CO_2_. On day 5, non-adherent cells were collected and DCs were enriched by density centrifugation over OptiPrep (Axis-Shield PoC AS, Oslo, Norway). Analysis of collected cells by flow cytometry revealed that the DC population was >80% positive for MHC class-II, CD80 and CD86, and >70% positive for CD11c (data not shown).

To generate B16 lysate, B16 melanoma cells were collected using trypsin/EDTA solution (Mediatech, Inc., Herndon, VA), washed twice with PBS and resuspended at 30 x 10^6^ cells/ml in PBS. The cells were subjected to four cycles of rapid freeze and thaw exposures. DCs were pulsed with tumor lysates at a ratio of 1 DC to 3 cell lysate equivalents for 18-24 hours.

### Flow Cytometry

For flow cytometric experiments, the following anti-mouse antibodies were used: Fluorescein isothiocyanate (FITC)-conjugated anti-mouse I-A^b^, CD8, CD11b, CD14, CD86, Ly6C and NK1.1; phycoerythrin (PE)-conjugated anti-mouse CD4, CD11b, CD11c, CD18, CD40, Ly6G and MARCO; APC-conjugated anti-mouse CD3, Gr-1 and CCR7 (all from BD Biosciences, San Jose, CA).

For cell surface marker staining, cells were washed with flow buffer (0.01% NaN_3_, 2% fetal bovine serum in PBS) and Fcγ II/III receptor blocking was performed by purified anti-mouse CD16/32 antibody (BD Biosciences). The blocking antibody (1µg/1 x 10^6^ cells) was added and cells were placed on ice for 10 min. After the blocking procedure, antibodies (1µg/1 x 10^6^ cells) for cell surface staining were added into each sample and placed on ice for 30 min protected from light. After two additional washes with buffer solution, all cells were fixed with 1% paraformaldehyde. Data acquisition was performed by flow cytometry (FACScan or FACSCalibur, BD Biosciences) within 24 hours after sample fixation. Data analysis was performed with CellQuest software (BD Biosciences).

### ELISA

To measure cytokine secretion, day 5 DC were not pulsed, cultured with B16 tumor lysate, or cultured with LPS. After 24 hours, culture supernatants were harvested for measurement of cytokine production by standard ELISA (BD Biosciences). To measure IFN-γ release, spleens were collected from mice receiving PBS, WT DC or MARCO^-/-^ dC.T cells were purified, then restimulated with CM, WT DC or MARCO^-/-^ DC. Supernatants were collected 48 hours later and tested for IFN-γ release by standard ELISA (BD Biosciences).

### Microscopy

Tumor lysate was stained with PKH26 red according to the manufacturer’s protocol (Sigma-Aldrich, St. Louis, MO). After 24 hours, the cells were harvested and washed with PBS. Lysate-pulsed DCs were stained with rat anti-MARCO mAb followed by the staining with Alexa Fluor 594 chicken anti-rat IgG (Invitrogen Corp.). Lysate pulsed-DC were washed twice with PBS, fixed with 1% PFA, and spun onto glass slides by a Shandon Cytospin-2 (International Medical Equipment, Inc., San Marcos, CA) at 800 rpm for 5 min. The slides were mounted with Vectashield mounting medium containing DAPI according to the manufacturer’s instructions (Vector Laboratories, Inc., Burlingame, CA). Slides were viewed with a fully automated, upright Zeiss Axio-ImagerZ.1 microscope (Carl Zeiss MicroImaging, Inc., Thornwood, NY). Images were produced using the AxioCam MRm CCD camera and Axiovision version 4.5 software suite (Carl Zeiss MicroImaging, Inc.).

### *Migration* In Vitro and In Vivo

DC pulsed with tumor lysate were used as responders at a concentration of 3 x 10^6^ DC cells/ml. Assays were performed in 24-well plate format with 5 µm pore polycarbonate Transwell inserts (Corning Inc, NY). Secondary lymphoid tissue chemokine (SLC, CCL-21) was added to the lower chambers at 100 ng/ml in CM. DC was added to the top chamber at 100 µl and incubated at 37^o^C with 5% CO_2_ for 5 hours. A 1:5 dilution of the cells was also directly added to the lower chamber of two wells for determination of the input amount. The inserts were removed and the cells were harvested, washed twice in PBS and stained with MHC Class II (I-A^b^) and CD11c to detect the DC. To quantify the number of migrated cells, polystyrene beads (Bangs Laboratories, Fishers, IN) were added to all samples and analyzed by flow cytometry (FACScan or FACSCalibur, BD Biosciences). The number of cells in each sample was determined by the equation: (number of cell events/number of bead events) x 10^4^ beads/sample. The percentage of migration in each sample (% input) was determined by the equation: (number of cells in sample/(number of cells in input x 5)) x 100.

For *in vivo* determination of migration of DC, BM-DC were pulsed with tumor lysate, stained with PKH26 red, and 5 x 10^6^ cells were injected into WT mice. After 60 hours, the vaccine-draining lymph nodes were collected. For confocal microscopy, the lymph nodes were fixed with 3.7% formaldehyde. For flow cytometry analysis, cells were stained with MHC Class II (I-A^b^) and CD11c. Polystyrene beads were added to all samples and analyzed by flow cytometry (FACScan or FACSCalibur, BD Biosciences). The cell number was calculated according to the following: 200,000/counted micro beads x number of cells positive for MHC Class II, CD11c, and PKH26 red.

### Treatment of Established Subcutaneous Tumor

Wild type and MARCO^-/-^ mice received 1 x 10^5^ viable B16 melanoma cells subcutaneously (s.c.) in the right flank. Three days after tumor injection, mice were treated with 1 x 10^6^ DC pulsed with tumor lysate or PBS in the left flank. Additional DC treatments were given on days 6 and 9 for a total of 3 DC injections. Tumor sizes were measured every 2-3 days after tumor injection.

### Statistical Analysis

All results for continuous variables are reported as mean ± SEM. Statistical analysis was performed by one way t-test. P values of 0.05 or less were considered statistically significant. Tumor treatment models were analyzed with GraphPad Prism (GraphPad Software, San Diego, CA).

## Results

### Generation of MARCO^-/-^ Mice

**Figure 1:**
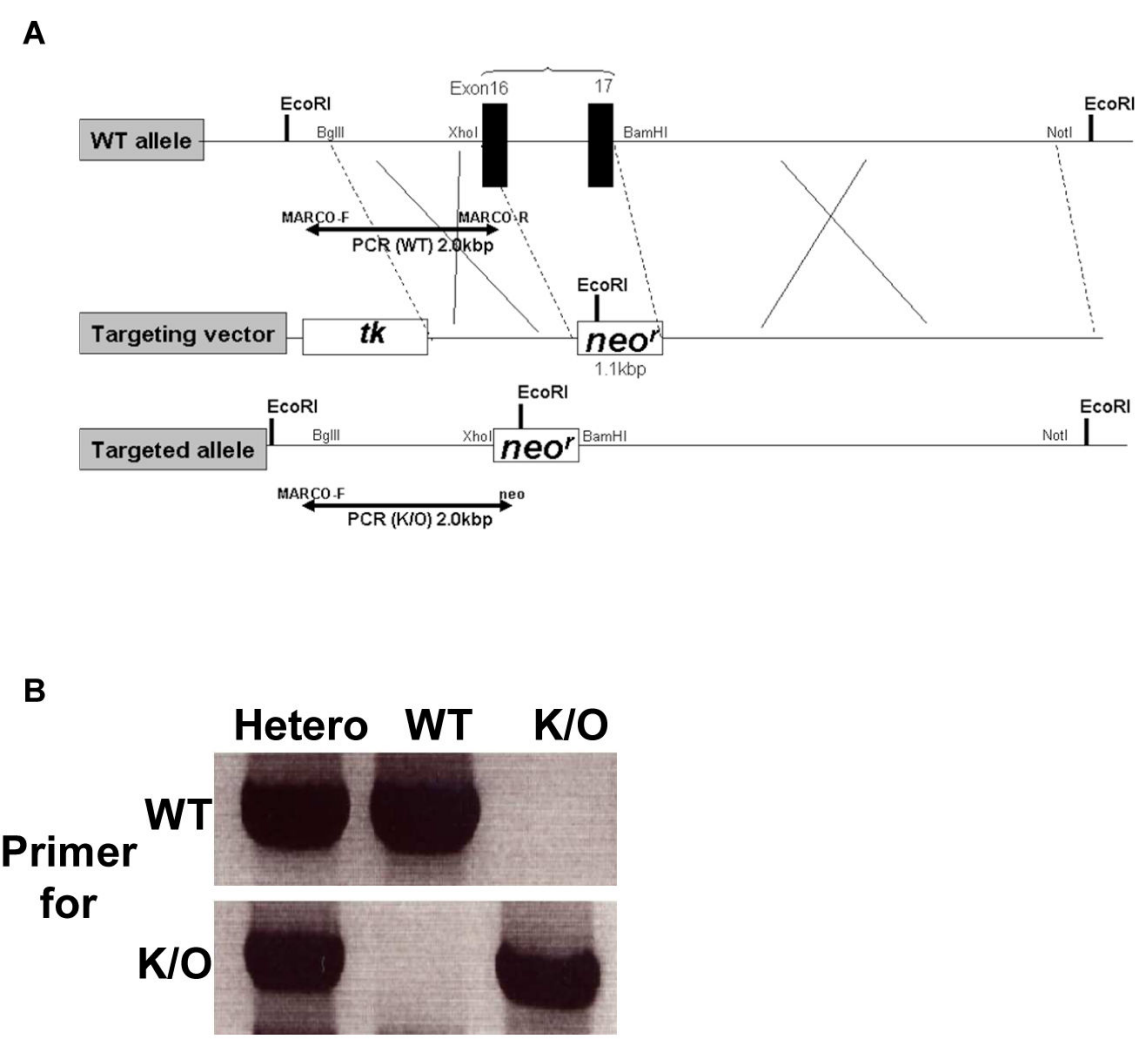
Development of a MARCO^-/-^ mouse. A) Schematic of the construction of MARCO ^-/-^ allele for the generation of MARCO^-/-^ mice. B) PCR analysis of MARCO gene expression in wild-type (WT) and knockout (K/O) mice.

A 228 bp probe containing exons 16 and 17 of the MARCO gene was used to screen a B6 LAMBDA DASH library (Stratagene, La Jolla, CA). As shown in Figure 1, the wild-type allele containing the MARCO gene was replaced using a targeting vector containing a Neomycin resistance (*neo* ^*r*^) gene. Briefly, the targeting vector was used to transfect B6 embryonic stem (ES) cells. Cells that had undergone homologous recombination with the targeting vector were resistant to both neomycin and acyclovir. Cells that randomly inserted the targeting vector were resistant to neomycin but sensitive to acyclovir. Gene insertion in positive clones selected in both neomycin and acyclovir was verified by PCR and Southern Blot (not shown). Positive ES clones were selected and micro-injected into albino C57BL/6 blastocysts. Blastocysts were implanted into pseudo-pregnant B6 females. Chimeras were bred to B6 albino mice and black offspring were screened by PCR to determine if the MARCO gene exons 16-17 had been deleted. Mice heterozygous for the MARCO gene knockout were bred to generate a homozygous strain of MARCO knockout mice (MARCO^-/-^). Figure 1B shows verification of the absence of the MARCO gene at the DNA level. MARCO gene knockout was also verified by Southern Blot analysis (not shown).

### MARCO Expression in DC

**Figure 2:**
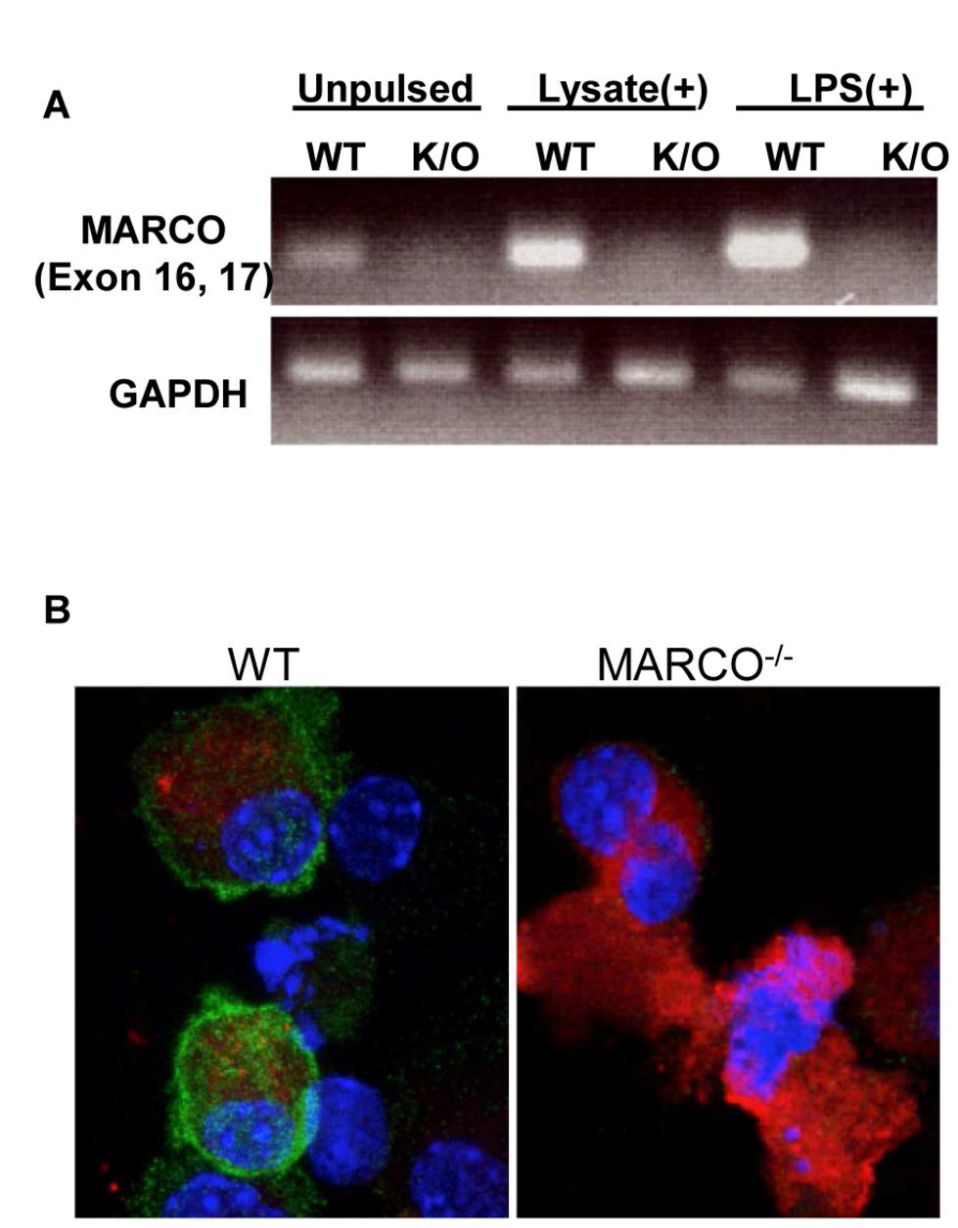
No MARCO expression is measured in DCs generated from the bone marrow of MARCO^-/-^ mice. A) RT-PCR analysis of MARCO expression in DCs generated from the bone marrow of wild-type or knockout mice. DCs were unpulsed, pulsed with B16 tumor lysate, or pulsed with 1 µg/ml LPS for 24 hours. Amounts of mRNA were adjusted to give comparable GAPDH signals. B) DCs from wild-type or knockout mice were pulsed for 24 hours with PKH26-red labeled tumor lysate (red). DCs were stained for MARCO expression (green) and DAPI (blue).

We first compared the expression of MARCO in DCs generated from the bone marrow of wild-type (WT) and MARCO^-/-^ (K/O) mice by gene expression analyses. As shown in Figure 2A, WT DCs express MARCO and MARCO expression is upregulated after exposure to either tumor lysate or LPS. Expression of MARCO in MARCO^-/-^ DCs was absent, even after pulsing with tumor lysate or LPS. Figure 2B confirms the loss of MARCO cell surface expression in DCs generated from MARCO^-/-^ mice. DCs from WT and MARCO^-/-^ mice were pulsed with tumor lysate, stained, and examined by microscopy.

### Comparison of Cell Surface Marker Expression in Lymph Node, Spleen, and DCs

**Figure 3:**
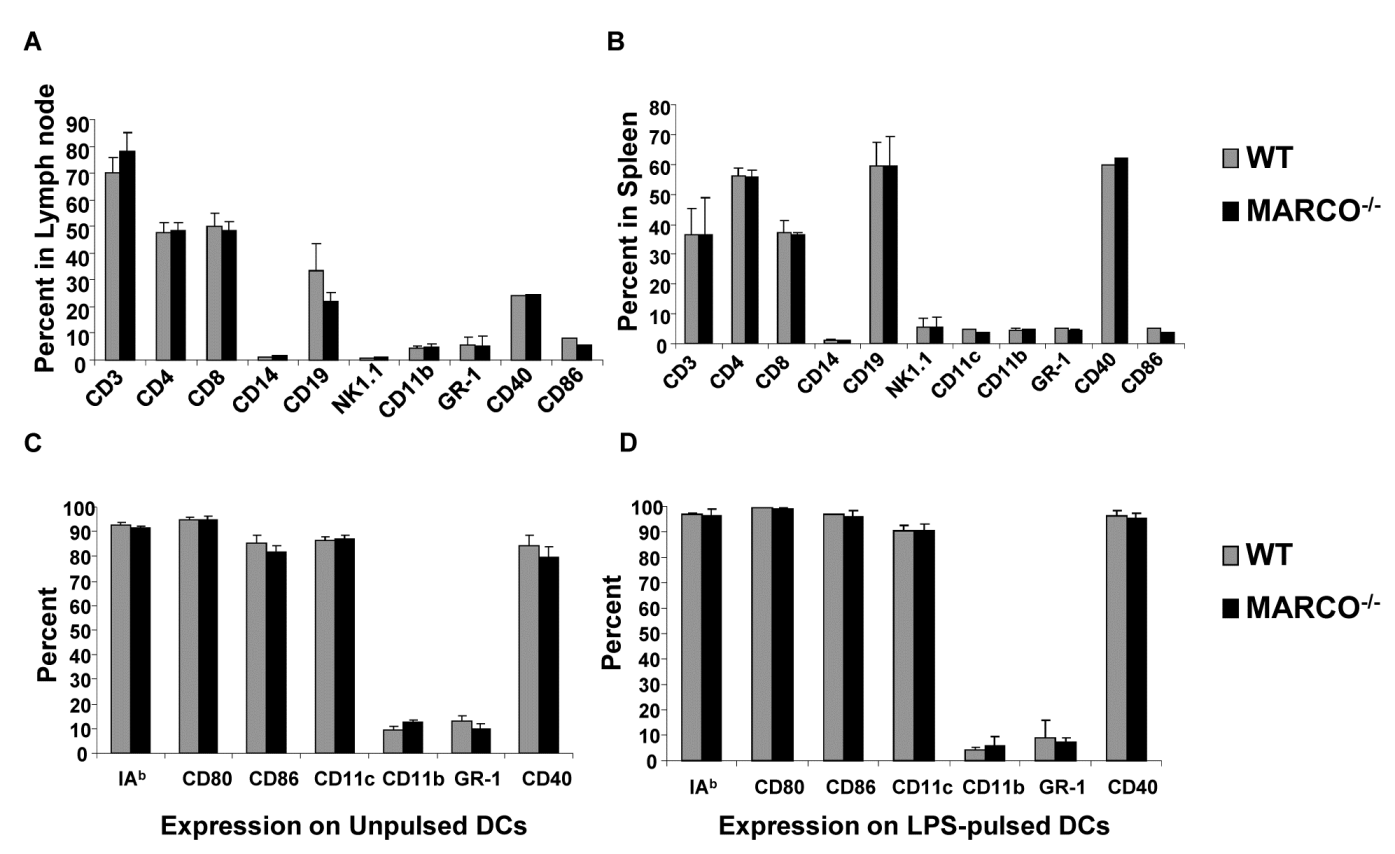
No difference in immune cell subsets or expression markers in WT and MARCO^-/-^ mice. Comparison of cell subsets in the (A) spleens and (B) lymph nodes of WT and MARCO^-/-^ mice. Comparison of cell surface markers on (C) unpulsed bone marrow-derived DCs and (D) DCs pulsed with LPS. Data shown is the average of three mice.

Lymph node cells (Figure 3A) and splenocytes (Figure 3B) from WT and MARCO^-/-^ mice were compared for surface expression levels of a selected battery of ancillary immune markers by flow cytometry. In contrast to MARCO expression, there were no significant differences detected between them. DCs generated from the bone marrow of WT and MARCO^-/-^ mice were unpulsed (Figure 3C) or pulsed with LPS (Figure 3D) or tumor lysate (not shown). No difference in expression levels of co-stimulatory molecules on the surface of DCs was measured.

### Comparison of Cytokine Production by DCs

**Figure 4:**
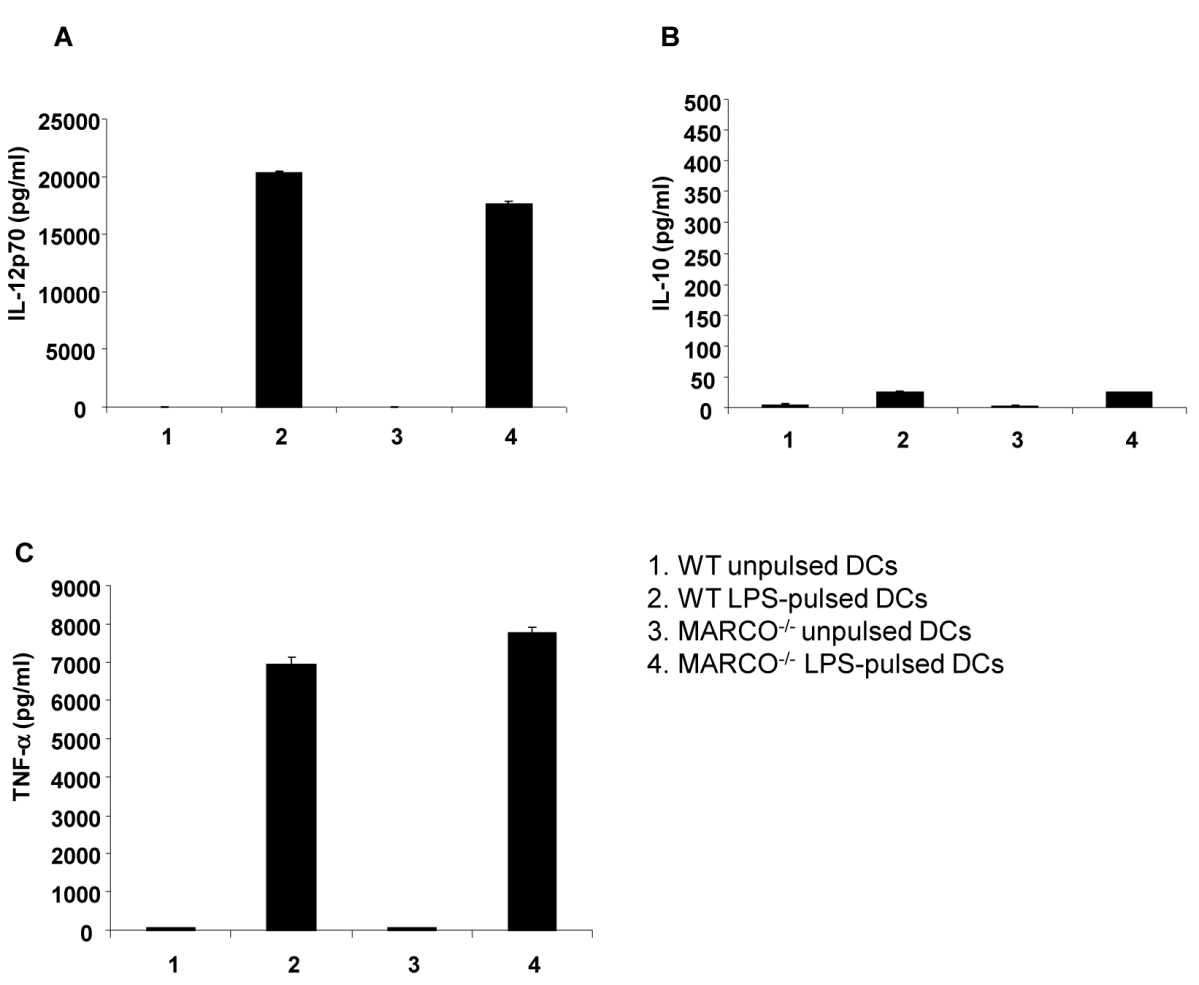
DCs generated from MARCO^-/-^ mice respond to LPS. Cytokine production by DCs generated from the bone marrow of WT or MARCO^-/-^ mice. The production of (A) IL12p70, (B) IL-10 and (C) TNF-α was measured. Experiments were repeated two times.

We also compared cytokine production of IL-12, IL-10 and TNF-α by unpulsed or LPS-pulsed DCs from WT and MARCO^-/-^ mice. As shown in Figure 4, the levels of cytokine production measured were comparable.

### Comparison of In Vitro and In Vivo Migration of DC

**Figure 5:**
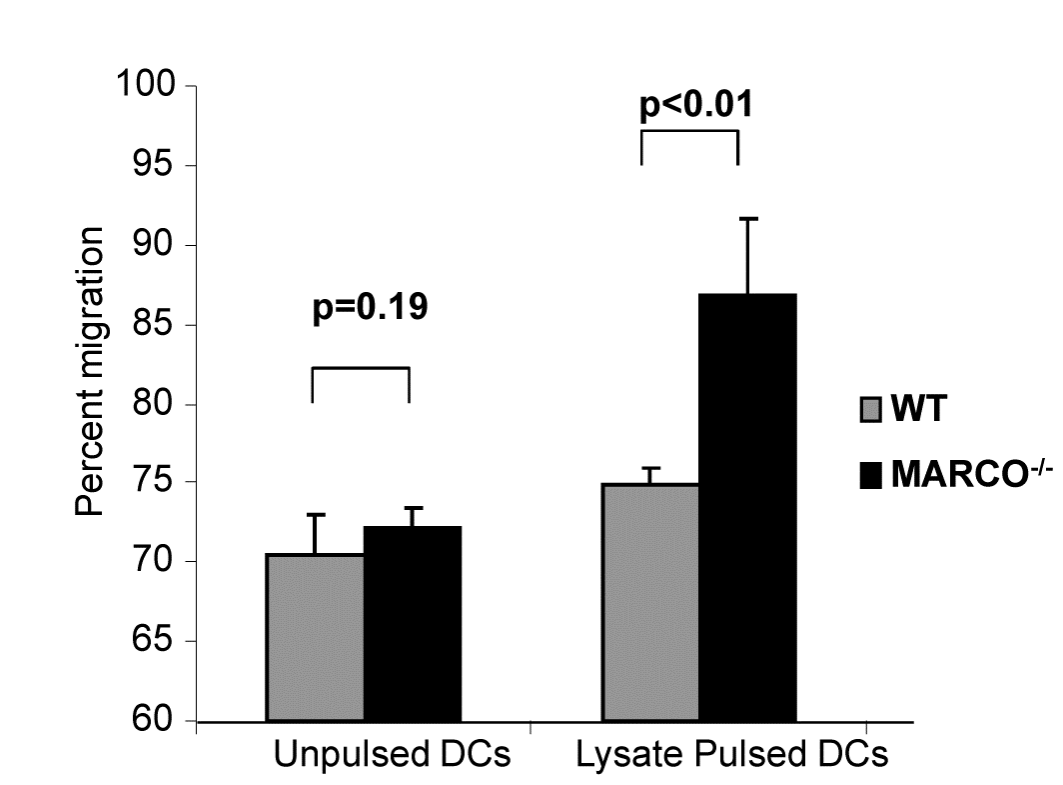
DCs generated from MARCO^-/-^ mice demonstrate enhanced capacity for *in vitro* migration. WT and MARCO^-/-^ DCs were unpulsed or pulsed with B16 tumor lysate. Cells were added to the upper chamber of a transwell plate. Recombinant CCL-21 (100 ng/ml) was added to the lower chamber. After five hours, cells in the lower chamber were collected and analyzed by flow cytometry. The percent migration of IA^b^ and CD11c double positive cells was calculated.

We next examined the effect, if any, of loss of MARCO on DC migration *in vitro*. DCs were unpulsed or pulsed with tumor lysate before their use in a micro-chemotaxis assay. DC migration was evaluated using the CCR7 ligand, CCL21, which is a known chemokine for DC migration. As shown in Figure 5, MARCO^-/-^ DCs that were pulsed with tumor lysate demonstrated a significantly higher migratory ability than WT DC. WT DC and MARCO^-/-^ DC expressed the same level of CCR7 expression on their respective cell surfaces (data not shown).

**Figure 6:**
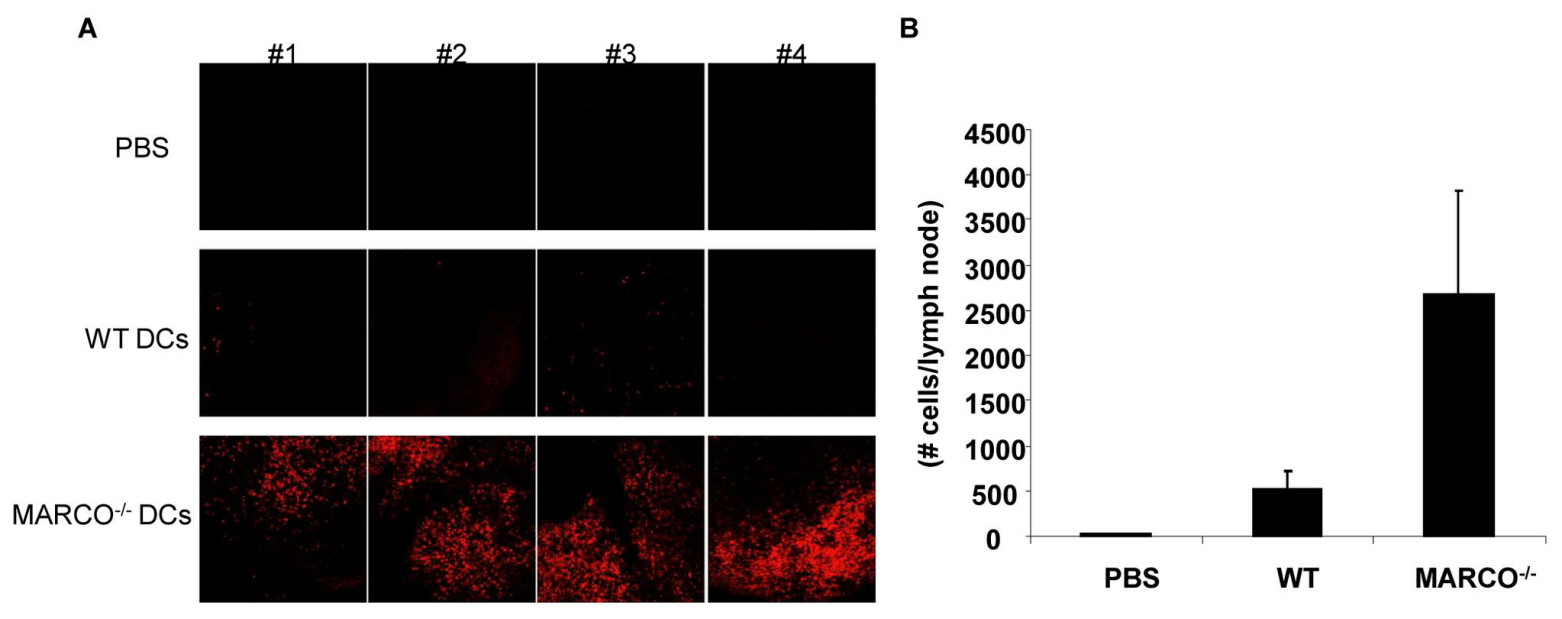
DCs generated from MARCO^-/-^ mice demonstrate enhanced capacity for migration *in vivo*. WT and MARCO^-/-^ DCs pulsed with B16 tumor lysate were stained with PKH26 red and injected s.c. to WT mice. After 60 hours, the draining lymph node was collected. (A) PKH26-red^+^ cells were visualized by microscopy (n=4 per group). (B) Lymph nodes were collected and dissociated into a single cell suspension. PKH26-red^+^ cells were quantified by flow cytometry. Data show as mean ± SEM of three mice per group.

For *in vivo* analysis, DCs from WT and MARCO^-/-^ mice were stained with PKH26 red dye and injected s.c. into WT mice. The draining lymph nodes were collected 60 hours later and examined for DC accumulation by confocal microscopy. As shown in Figure 6, the lymph nodes of WT mice injected with MARCO^-/-^ DCs showed a dramatic accumulation of these injected cells, which was confirmed by flow cytometry (Figure 6B, p<0.05 WT compared to K/O). In this latter study, single cell suspensions of the draining lymph nodes were prepared, depleted of erythrocytes, stained with MHC Class II and CD11c antibody, and microbeads added to enumerate the number of cells infiltrating the lymph node.

### IFN-γ Production by Stimulated T Cells

**Figure 7:**
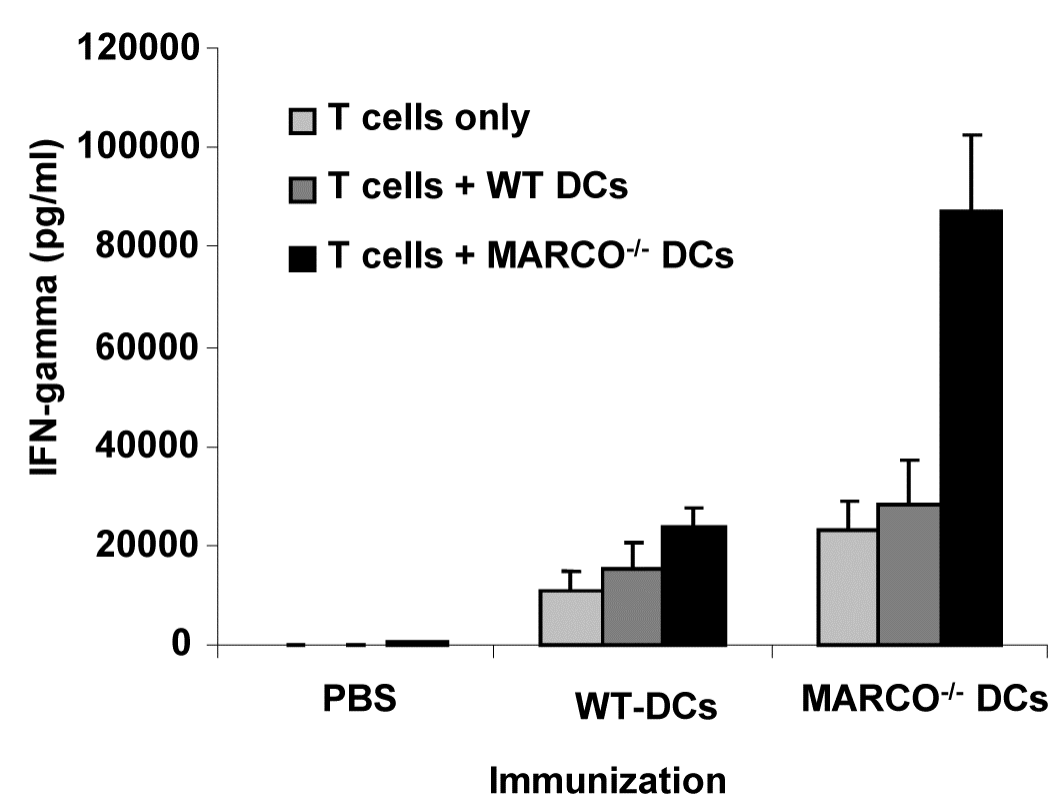
WT mice were vaccinated with lysate pulsed-DCs derived from the bone marrow of WT or MARCO^-/-^ mice on days 0, 3, and 6. On day 13, spleens were collected and T cells were purified using a T cell enrichment column. T cells were incubated with irradiated WT or MARCO ^-/-^ DCs at a ratio of 10 T cells : 1 DC. After 48 hours, supernatants were collected and IFN-γ production was measured by ELISA. Each data shows the average of three mice.

We also examined the capacity of antigen loaded DCs to effectively prime T cells *in vivo* following s.c. injection. As shown in Figure 7, T cells from WT mice immunized with tumor lysate-pulsed MARCO^-/-^ DCs and then restimulated *in vitro* with tumor lysate-pulsed MARCO^-/-^ DCs produced the highest levels of IFN-γ (p<0.001 compared to all other groups).

### Treatment of Established B16 Melanoma with WT and MARCO^-/-^ DCs

**Figure 8:**
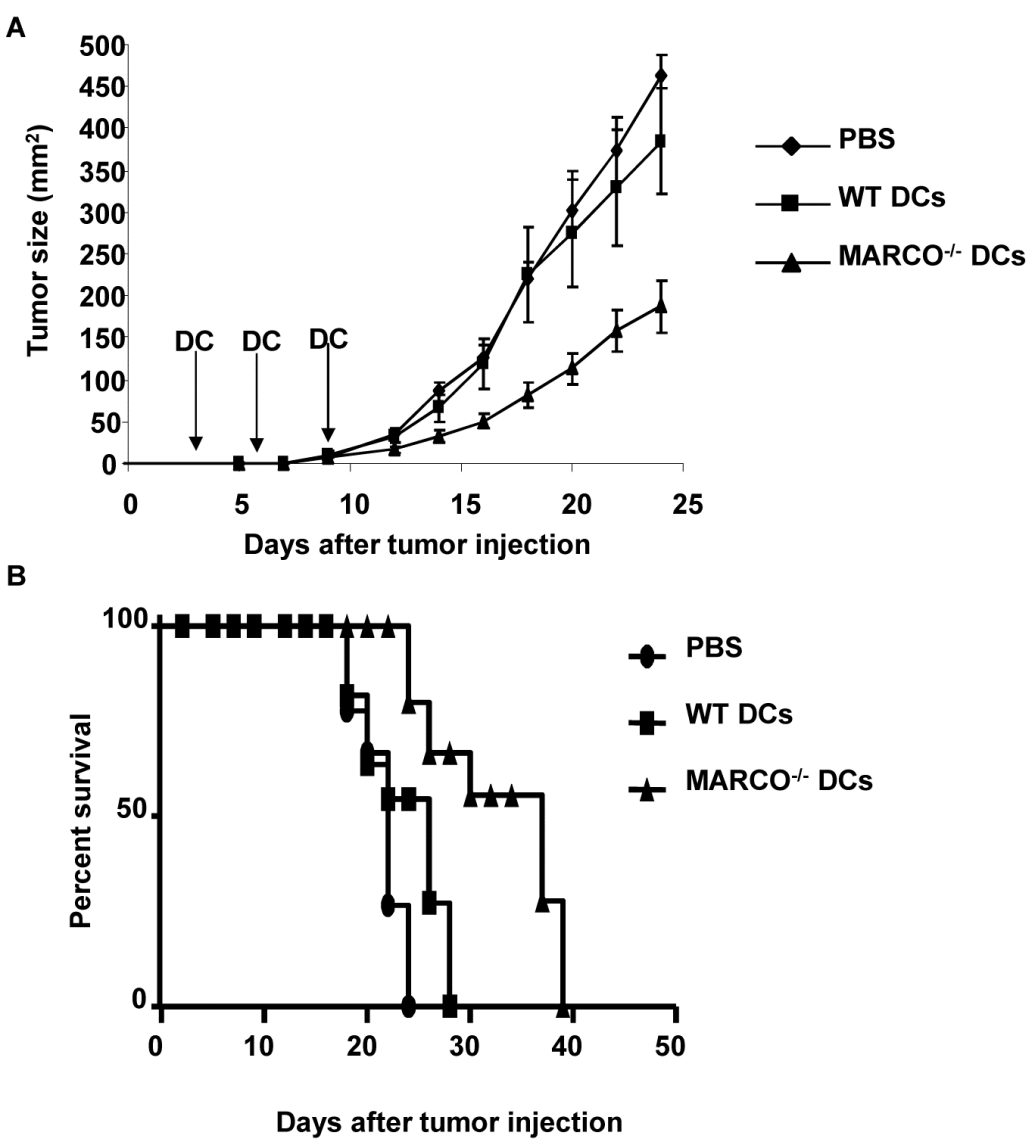
Mice were injected s.c. with B16 cells on day 0. Mice were immunized s.c. with 1x10^6^ DCs on days 3, 6, and 9 with B16 tumor lysate pulsed WT or MARCO^-/-^ DCs or PBS. (A) Tumor growth and (B) survival were measured. n=7-8 mice per group. Experiment was performed two times with similar results.

To test the therapeutic efficacy of MARCO^-/-^ DC treatment on tumor growth, we first injected B16 tumor cells s.c. into C57BL/6 mice. Mice were then vaccinated on days 3, 6 and 9 with WT or MARCO^-/-^ DCs. As shown in Figure 8A, mice treated with MARCO^-/-^ DCs demonstrated delayed tumor growth (p<0.05 compared to other treatment groups). These mice also exhibited prolonged survival as compared with the mice that received WT DCs or PBS (Figure 8B, p<0.05 compared to other groups).

## Discussion

The current study has shown that loss of expression of the scavenger receptor MARCO enhances the trafficking and anti-tumor efficacy of DCs pulsed with tumor lysates. DCs generated from the bone marrow (bm) of MARCO^-/-^ mice were phenotypically identical to DCs generated from the bm of wild-type mice. DCs expressed high levels of MHC and co-stimulatory molecules and responded to LPS by producing IL-12 and TNF-α. No difference in phagocytic capacity was measured between MARCO^-/-^ and wild-type DCs after 24 hours of lysate pulsing (not shown). MARCO^-/-^ DCs were morphologically normal. This was surprising as MARCO has been implicated in actin cytoskeleton rearrangements and our previous studies demonstrated that targeting MARCO with a monoclonal antibody led to a loss of dendritic-like processes on the surface of DCs [11,14].

While the role of scavenger receptors in innate immunity is well documented, the role of these receptors in T cell priming and adaptive immunity is less defined. Scavenger receptors are pattern recognition receptors with broad ligand-binding specificities [15]. Scavenger Receptor A (SRA) and MARCO are members of the Class A Scavenger Receptor family and are expressed in macrophages and dendritic cells. Both act as phagocytic receptors of multiple ligands including pathogenic bacteria and apoptotic cells and participate in cell adhesion and migration [16–18]. While these receptors cannot initiate inflammatory responses by themselves, SRA and MARCO may play a role in attenuating innate immune responses to ligands [19,20]. Blockade of SRA and MARCO has been shown to enhance inflammation in response to LPS [21].

Recent studies have demonstrated the ability of scavenger receptors to modulate the induction of T cell responses [22–24]. It has been reported that vaccination with DCs generated from the bone marrow of SRA knockout mice leads to enhanced T cell priming and anti-tumor efficacy [25]. In our study, vaccination with tumor lysate pulsed, MARCO-deficient DC led to enhanced anti-tumor T cell activity and regression of B16 melanoma. We performed additional studies in which mice were vaccinated with tumor lysate-pulsed DC generated from the bone marrow of mice deficient in both MARCO and SRA. In these studies, there were no differences in anti-tumor T cell activity or tumor regression observed between vaccination with MARCO^-/-^ DCs and the double knock-out DCs (not shown). These results may indicate a redundant role of SRA and MARCO in DCs for the induction of adaptive immunity.

Vaccination with tumor antigen-pulsed DCs has been shown to be safe and effective at inducing anti-tumor T cell responses in patients with advanced cancers, although these responses rarely translate to clinical efficacy. As injected DCs must migrate to a draining lymph node to stimulate antigen-specific CD4^+^ and CD8^+^ T cells, strategies to improve the migration of DCs have been explored. Intradermal injection of DCs results in small numbers of DC trafficking to draining lymph nodes with the majority of DCs localized to the vaccination site [26,27]. Direct intranodal injection of DCs to enhance anti-tumor T cell activation by increasing the number of DCs localized in the draining lymph node has also been explored. Several studies have shown that although higher numbers of DCs can be detected in the lymph node after intranodal injection, the quality of T cell responses were inferior to those induced by intradermal vaccination of DCs [28–30]. It has been suggested that migration of DC is required for maximum maturation and potency resulting in the induction of strong, durable anti-tumor immune responses [31]. We have previously shown that exposure of tumor lysate-pulsed DCs with an anti-MARCO antibody enhanced DC migration to draining lymph nodes [14]. In this study, we have verified that loss of MARCO expression in DC enhances migration *in vitro* and *in vivo*. We believe this enhanced migration led to the enhanced anti-tumor T cell activity and tumor regression that we observed.

Our studies have confirmed the role of MARCO on the migration of injected DCs from a subcutaneous injection site to the draining lymph node. We have previously demonstrated the expression of MARCO on the surface of human monocyte-derived DCs [14]. Strategies to target MARCO on the surface of human DCs may improve responses in clinical trials employing DC-based vaccination.
